# Supplementary material for: Mechanisms of activation and desensitization of full-length glycine receptor in lipid nanodiscs
Source: Nat Commun. 2020 Jul 27;11:3752. doi: 10.1038/s41467-020-17364-5 (PMC7385131; doi:10.1038/s41467-020-17364-5)
Supplement: Supplementary file 1 — Supplementary Information [file 41467_2020_17364_MOESM1_ESM.pdf]

Supplementary Information

# Mechanisms of activation and desensitization of full-length glycine receptor in lipid nanodiscs

Kumar et al.

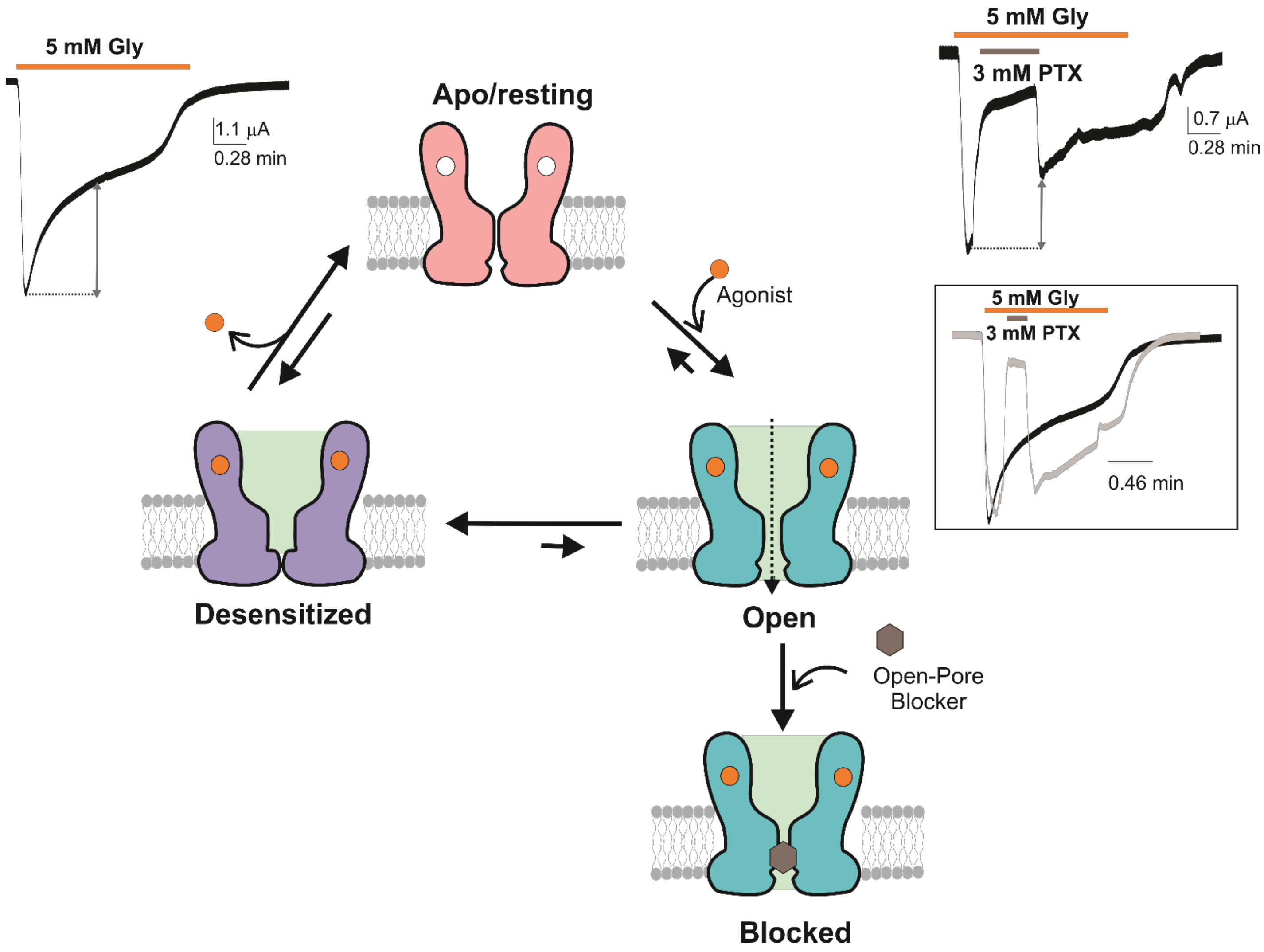


**Supplementary Figure 1. GlyR gating cycle.** Schematic representation of GlyR gating involving transitions from the Apo (resting) to glycine-bound, open and desensitized conformations. PTX, an open-channel pore blocker, inhibits glycine-evoked currents. Two-electrode voltage clamp (TEVC) recording of GlyR expressed in *Xenopus* oocytes. Currents were elicited in response to application of 5 mM Glycine with a holding membrane potential of -60 mV. Robust inward currents are observed that desensitize in the presence of glycine (n = 10) *(Left)*. A TEVC recording showing the effect of 3 mM PTX block on GlyR current evoked by application of 5 mM glycine with a holding membrane potential of -60 mV (representative trace from n = 5). Upon wash-off of PTX, the amplitude of recovered currents (indicated by arrows) reflects the population of channels still conductive *(Right)*. Inset shows an overlay of representatives trace with and without PTX normalized to peak amplitude. n is individual oocyte.

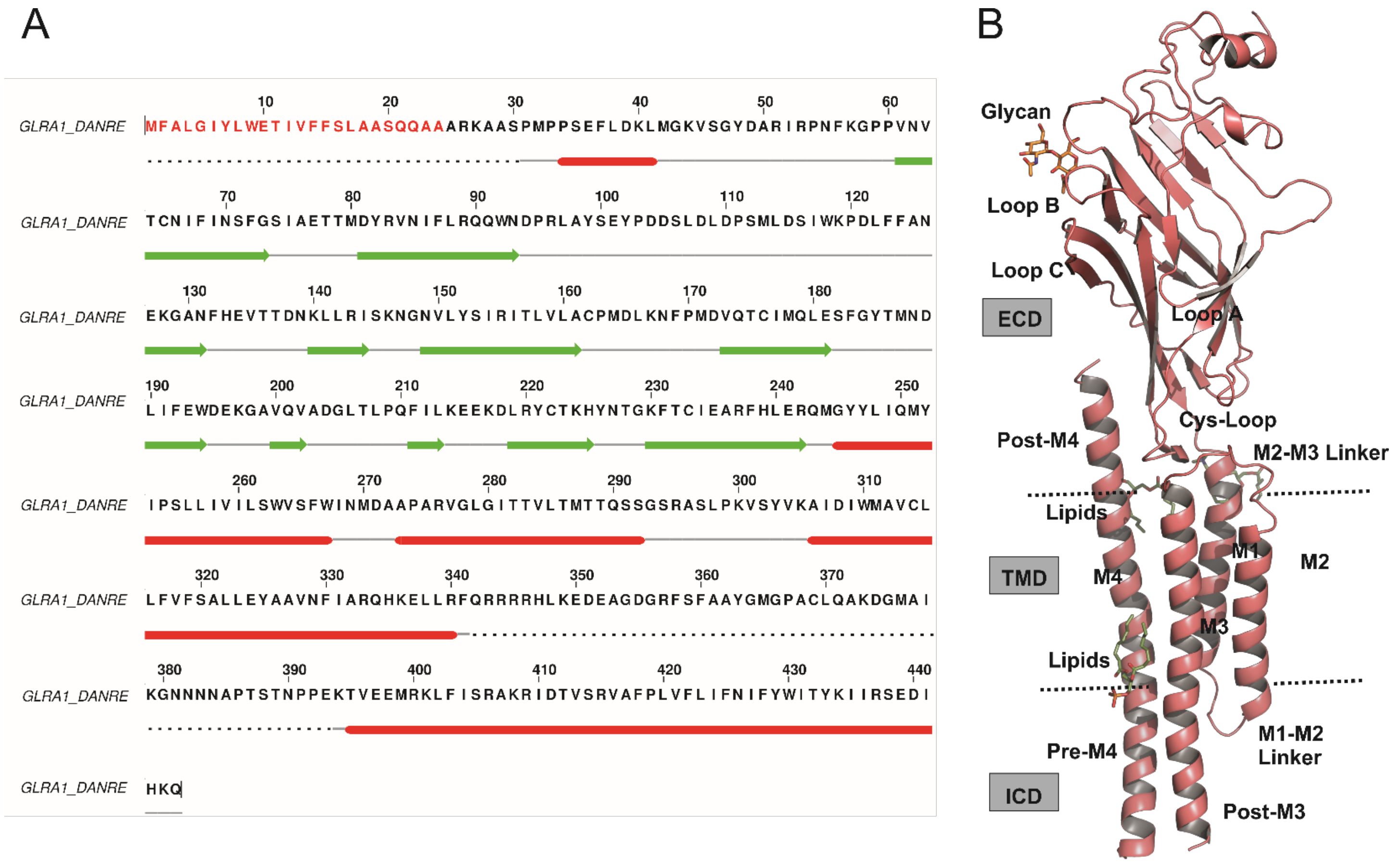


**Supplementary Figure 2. Sequence and topology of GlyR** A) Sequence of zebrafish GlyRα1 used in the cryo-EM study and electrophysiological analysis. Secondary structural elements are indicated below the sequence. Dotted line denotes the residues not included in the GlyR-Apo model. B**)** A single-subunit of GlyR-Apo, viewed from a plane parallel to the membrane, with secondary structure elements labeled. The glycans are shown as orange sticks and lipids are shown in green sticks. The putative membrane limits are marked by dotted lines.

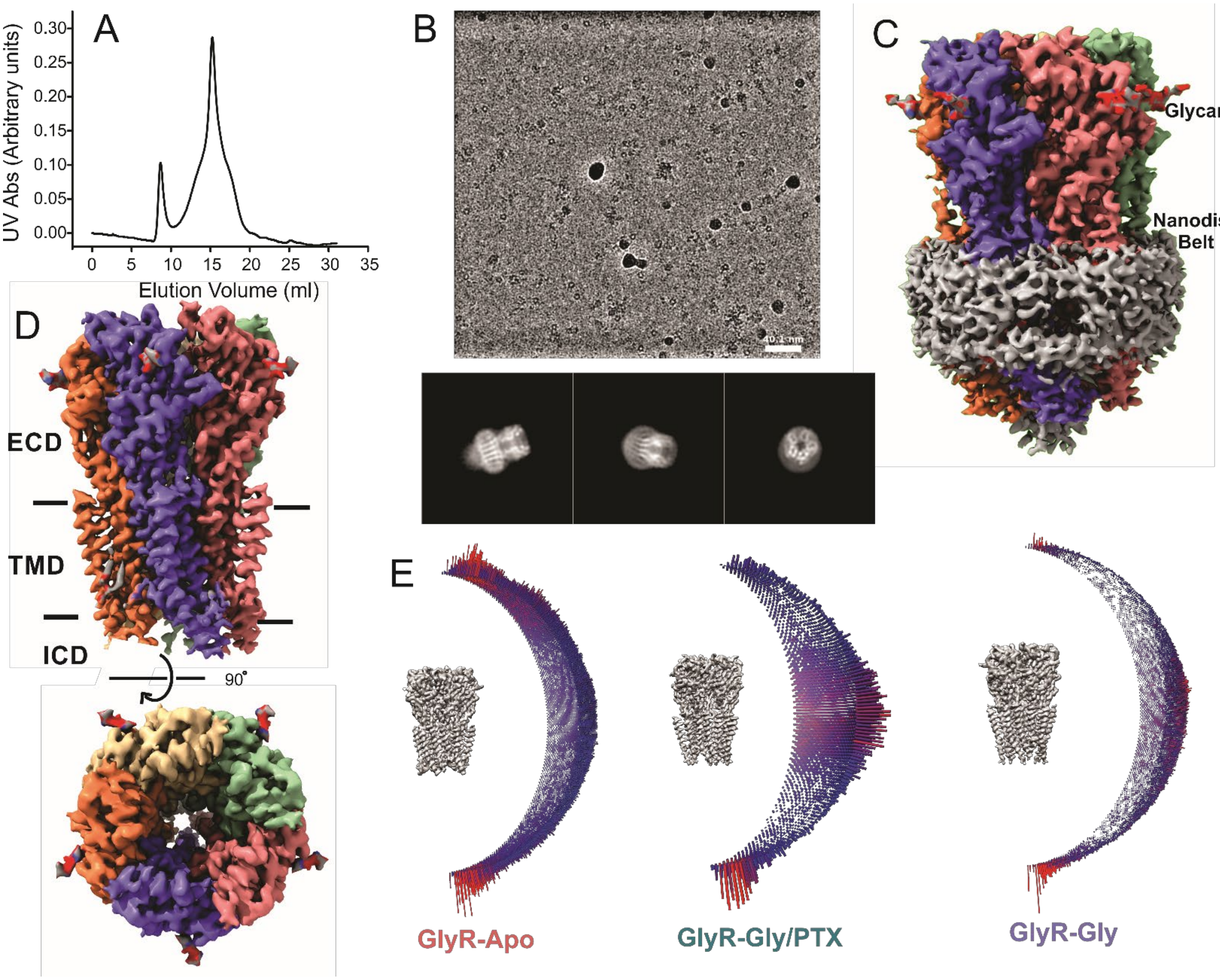


**Supplementary Figure 3. Biochemical and structural characterization of GlyR** A) Gel-filtration profile of affinity-purified GlyR on a Superose 6 Increase 10/300 GL column (GE Healthcare). The main peak corresponds to the GlyR pentamers. B**)** A representative cryo-EM micrograph of nanodisc-reconstituted GlyR-Apo sample in vitreous ice (*top*) and selected 2D classes showing various orientations (*bottom*)**.** GlyR samples were imaged multiple times under different conditions, the quality of the micrographs and the 2D classes were comparable. C**)** Cryo-EM 3D reconstruction of GlyR-Apo. Each subunit is individually colored for clarity and the glycans are show in red. The density corresponding to the nanodisc belt is colored gray. D) Side and top views of the 3D reconstruction showing GlyR-Apo after subtracting the nanodisc belt. The individual domains are marked. E**)** Angular distribution of particle projections for the final reconstruction used for model building. The map of the GlyR-Apo, GlyR-Gly and GlyR-Gly/PTX complex is shown in gray. Nanodisc belts have been removed for clarity. Length of each cylinder corresponds to the number of particles at a specific Euler angle.

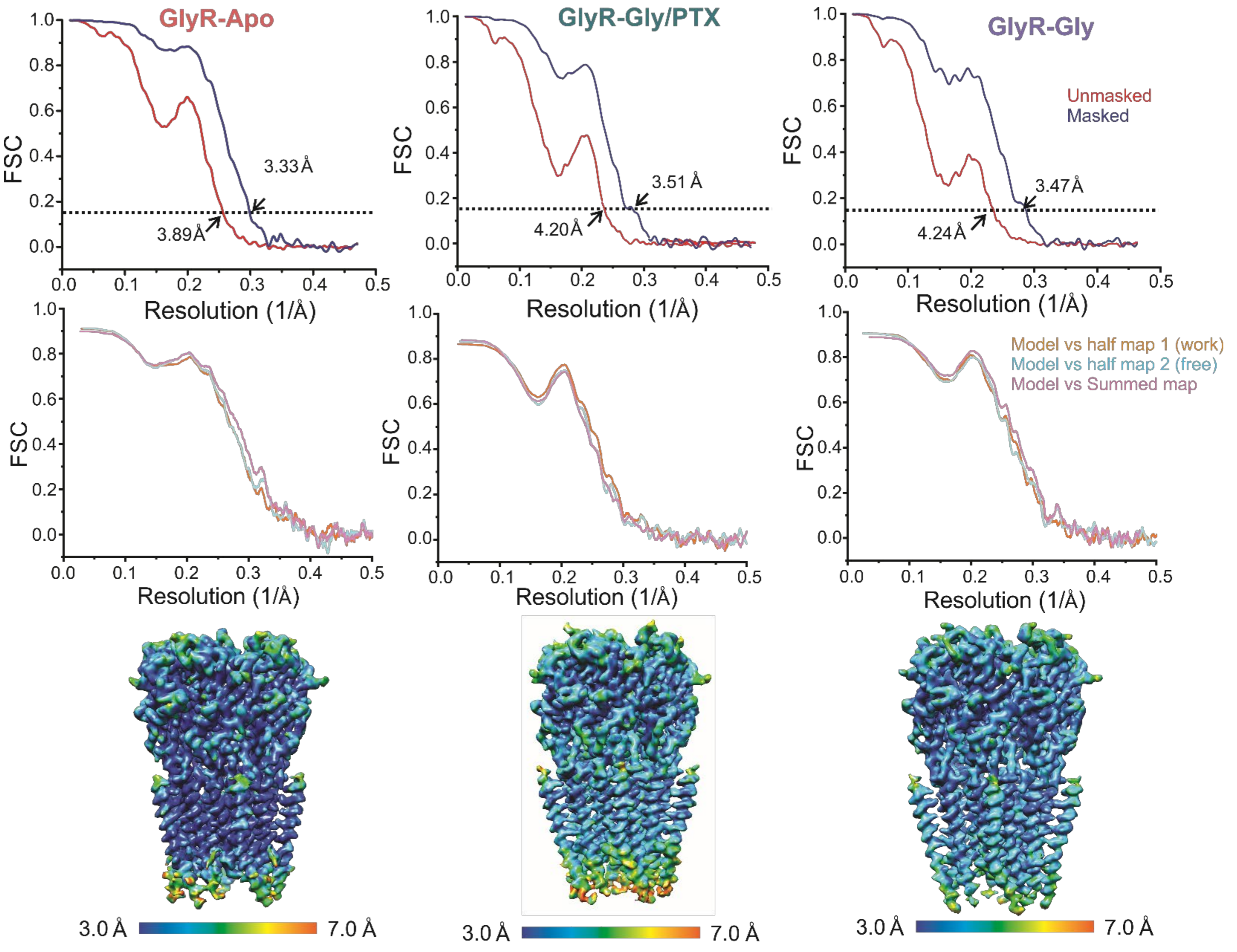


**Supplementary Figure 4. Resolution estimation and model validation.** Gold standard Fourier shell correlation (FSC) curves RELION 3.0 *(top)*. The dashed line represents an FSC of 0.143. For cross validation of model refinement, FSC curves of the refined model versus summed map (full dataset), refined model versus half map 1 (used during refinement), and refined model versus half map 2 (not used during refinement) *(middle)*. Side views of the 3D reconstructions colored-coded by the local resolution determined using ResMap program algorithm v1.1.5 [1] *(bottom)*.

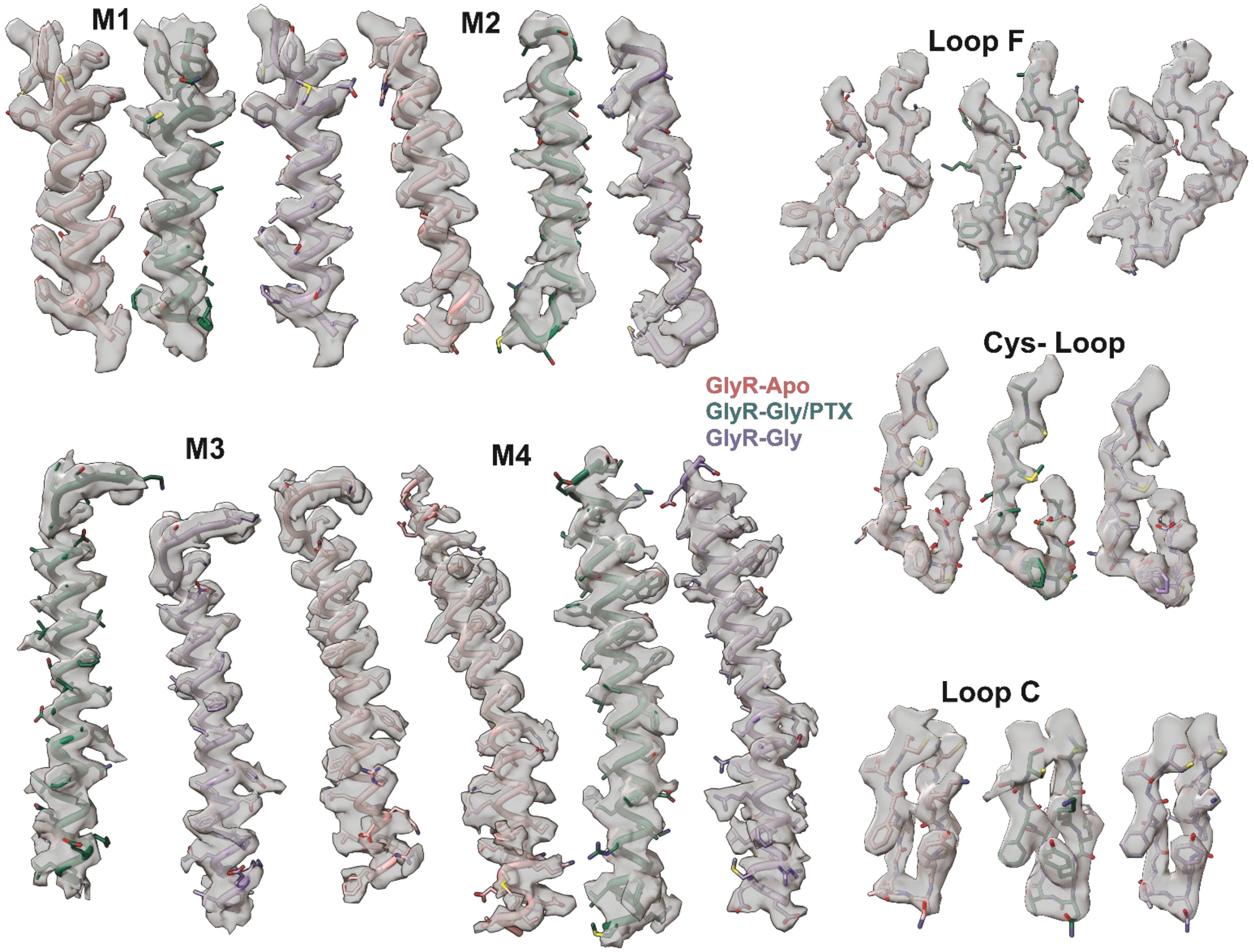


**Supplementary Figure 5. Map correlation of GlyR structures.** Validation of various regions within each of the domains of the model (shown as cartoon with stick representation for the residues) and corresponding density map (volume) are shown here. Density map for GlyR-Apo, GlyR-Gly, and GlyR-Gly-PTX was contoured at a threshold of 0.008, 0.010 and 0.009 respectively in UCSF ChimeraX (https://www.cgl.ucsf.edu/chimerax/).

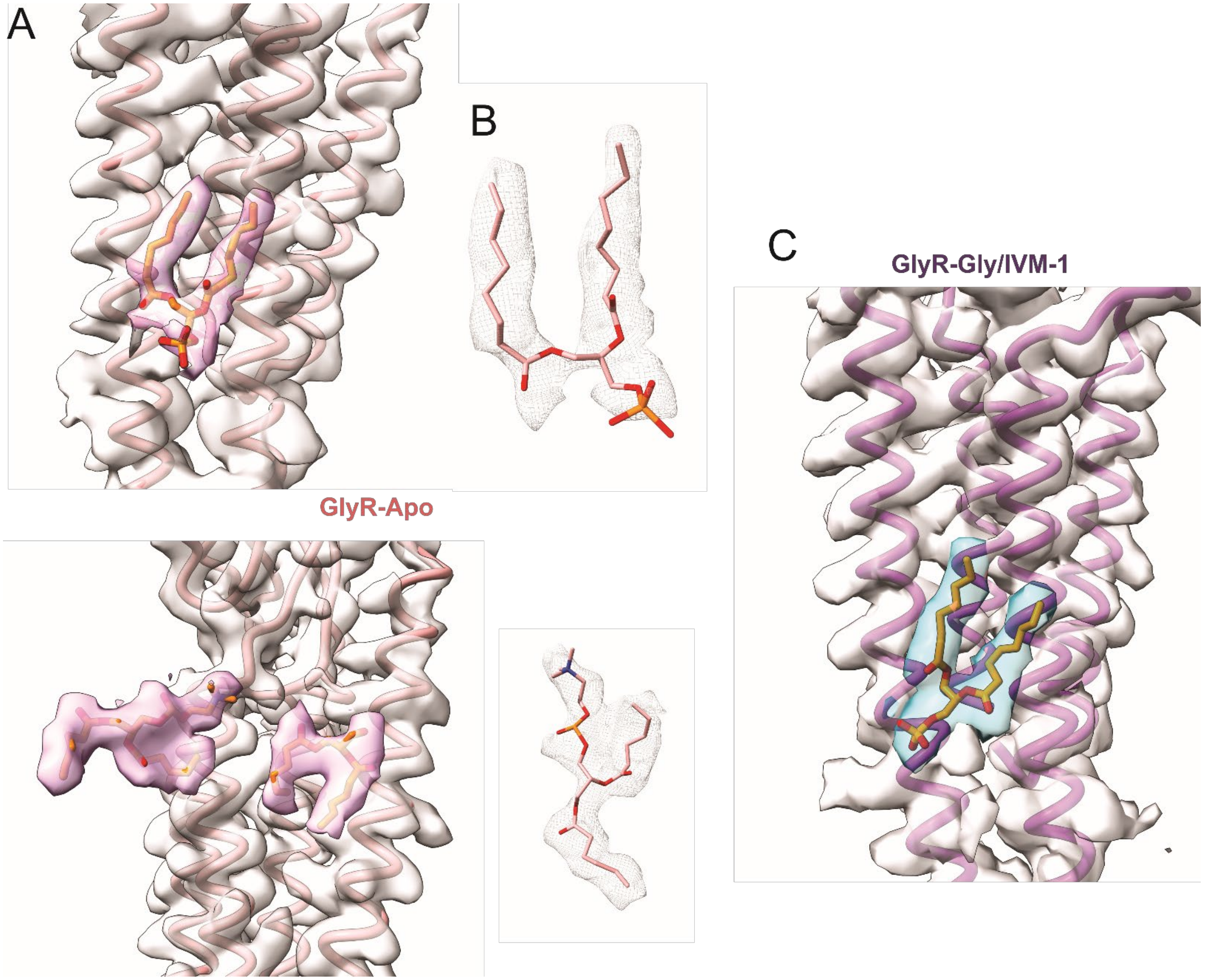


**Supplementary Figure 6. Lipid-like densities in the cryo-EM map.** A) GlyR-Apo density map revealing lipid-like density near the intracellular end of M4. The lipid and protein density are shown at the same contour. B) Lipid densities at the extracellular end of M4 in GlyR-Apo. C) A close up view of the lipid map and model. C) GlyR-Gly/IVM1 structure and the corresponding map for protein and the lipid at the intracellular end.

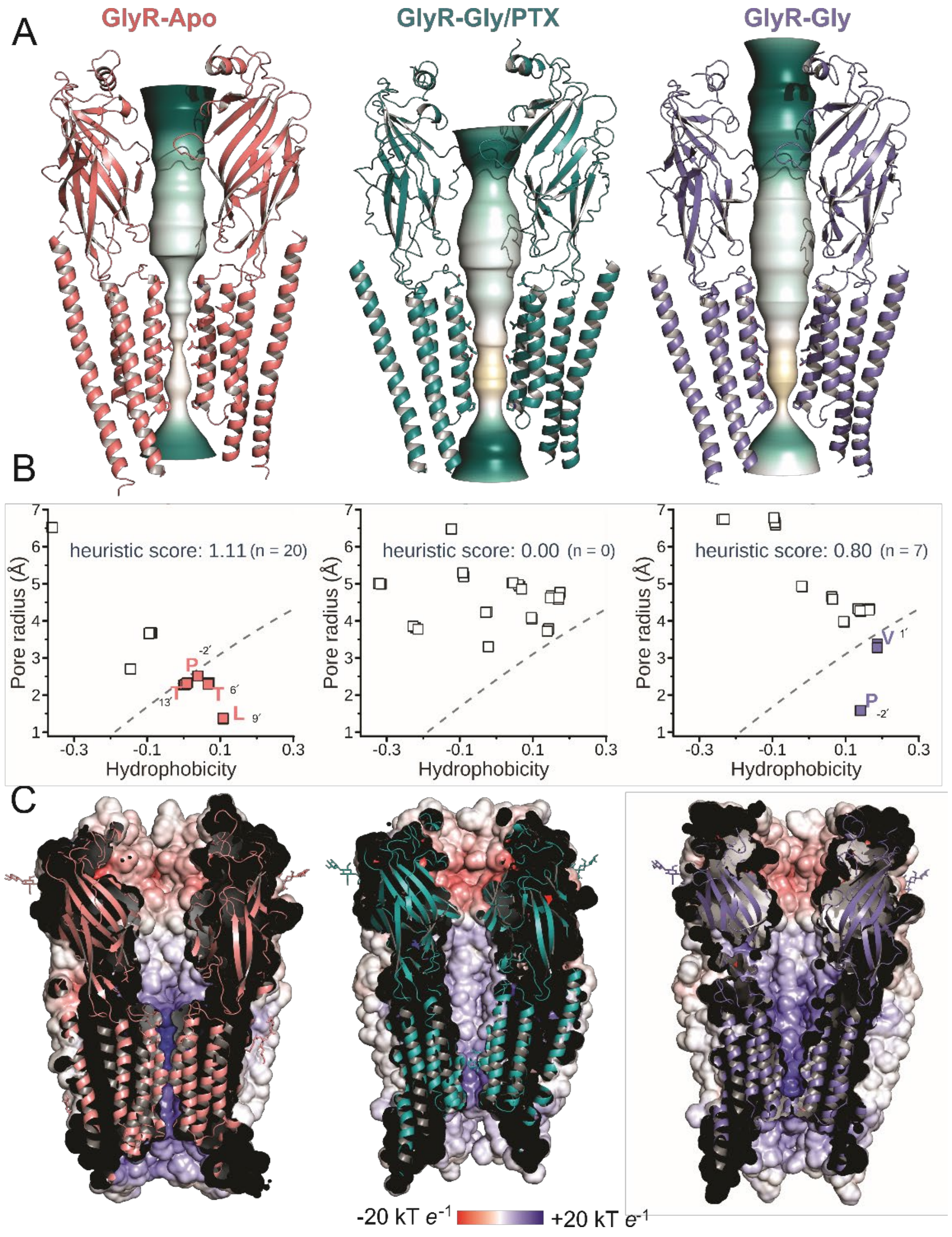


**Supplementary Figure 7. Assessment of pore properties.** A) Pore surface through each channel structure, colored by hydrophobicity (from hydrophilic-green, to hydrophobic-yellow) as estimated by CHAP[3] based on the experimental hydrophobicity values of pore-lining amino acid side-chains. B) Assessment of the likelihood of pore closure by an energetic barrier corresponding to dewetting at a hydrophobic region, evaluated according to a heuristic method based on simulation of water behavior in ~200 ion channel structures[2]. Any identified pore-lining sidechains are shown as points and were mapped onto the prediction grid with their local pore hydrophobicity and radius as coordinates. The subset of those falling below the dashed classification line were used to calculate a heuristic score. A cut-off of > 0.55 had been used to predict that a channel structure would contain a hydrophobic barrier to water and ion permeation. C) The vertical slice-through of the receptor showing the electrostatic surface potential generated using the APBS tool plug-in in PyMOL[3] along the ion-conducting pathway for GlyR-Apo, GlyR-Gly/PTX and GlyR-Gly conformations.

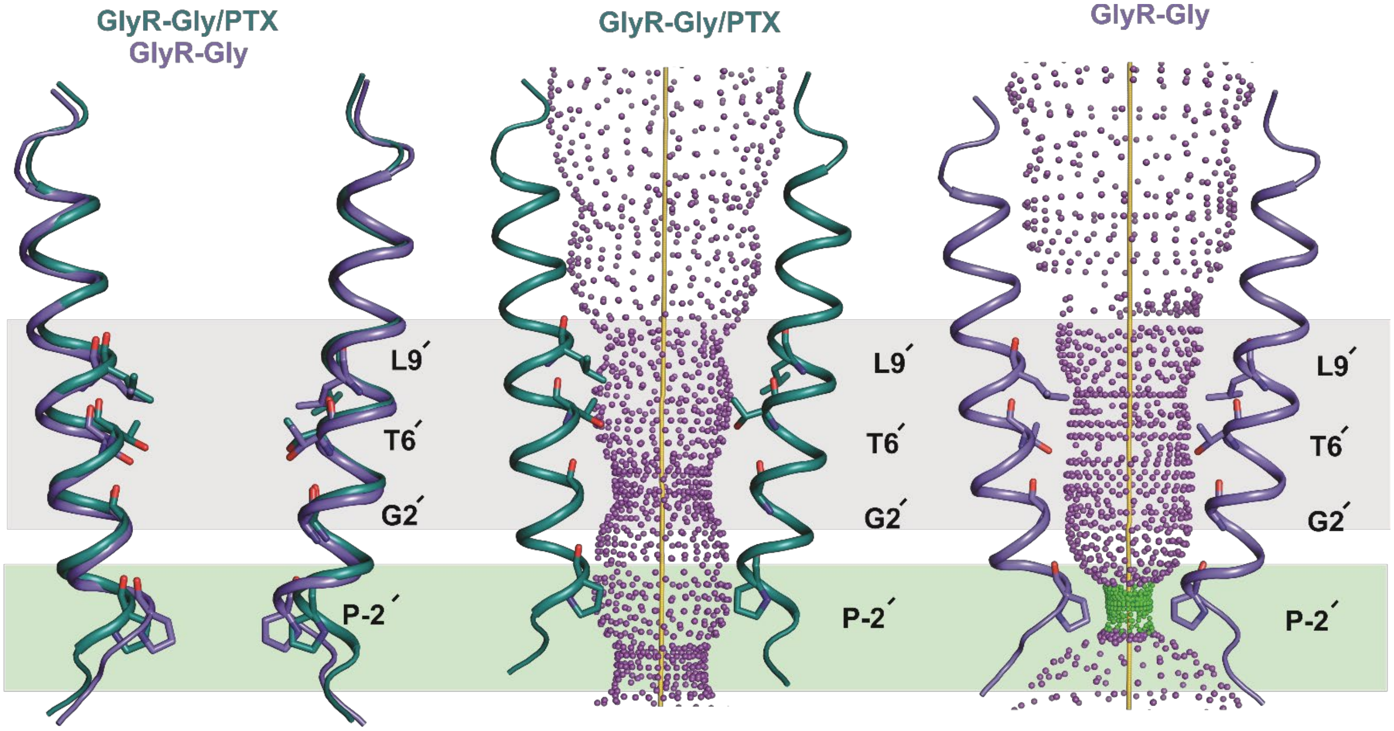


**Supplementary Figure 8. Comparison of GlyR-Gly and GlyR-Gly/PTX pores.** Alignment of M2 helices from GlyR-Gly/PTX and GlyR-Gly *(left)*. Pore profile of the GlyR-Gly/PTX and GlyR-Gly conformations *(right)*. The PTX binding site and the desensitization gate (Pro-2′) are highlighted gray and green boxes, respectively. Primary differences between the two structures are at the level of the desensitization gate.

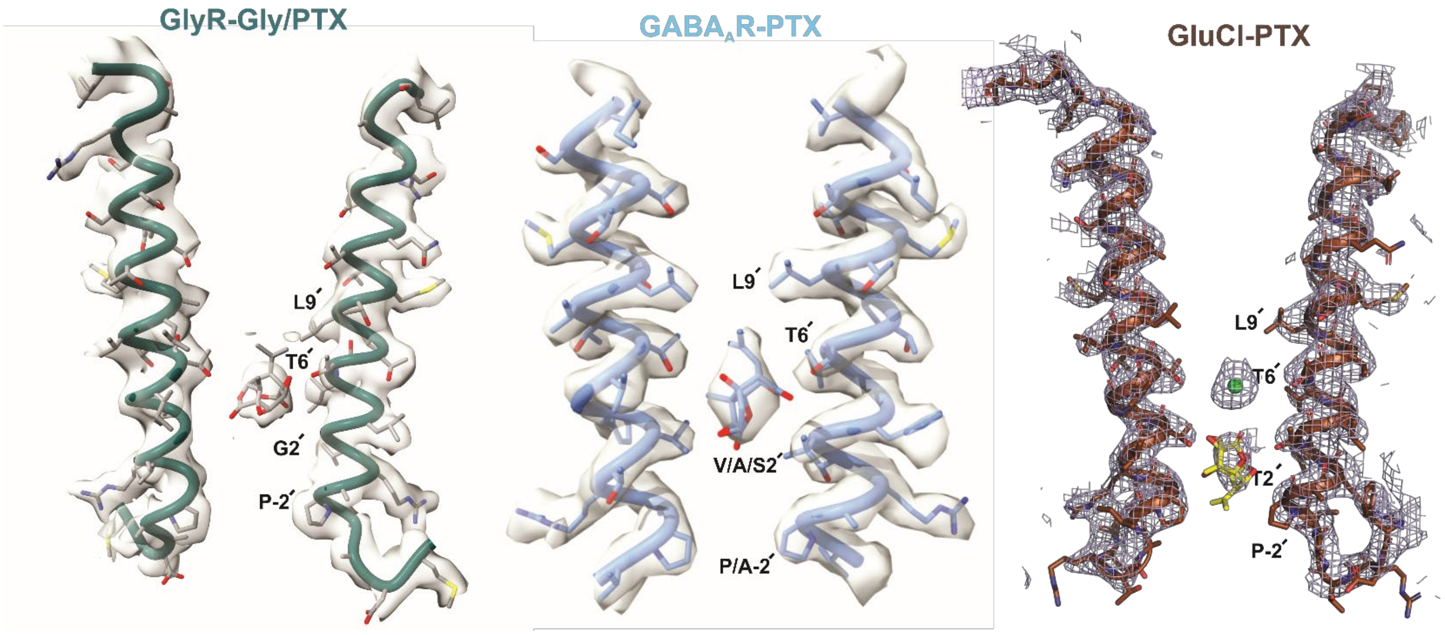


**Supplementary Figure 9. Comparison of PTX binding site in anionic pLGIC structures:** Side by side comparison of density map and model of M2 along with PTX in GlyR-Gly-PTX, α1β3γ2-GABA$_A$R-PTX (PDB_ID:6HUJ)[4] and GluCl-PTX (PDB_ID:3RI5) [5]. In the current structure, the PTX was modelled based on GABA$_A$R-PTX structure.

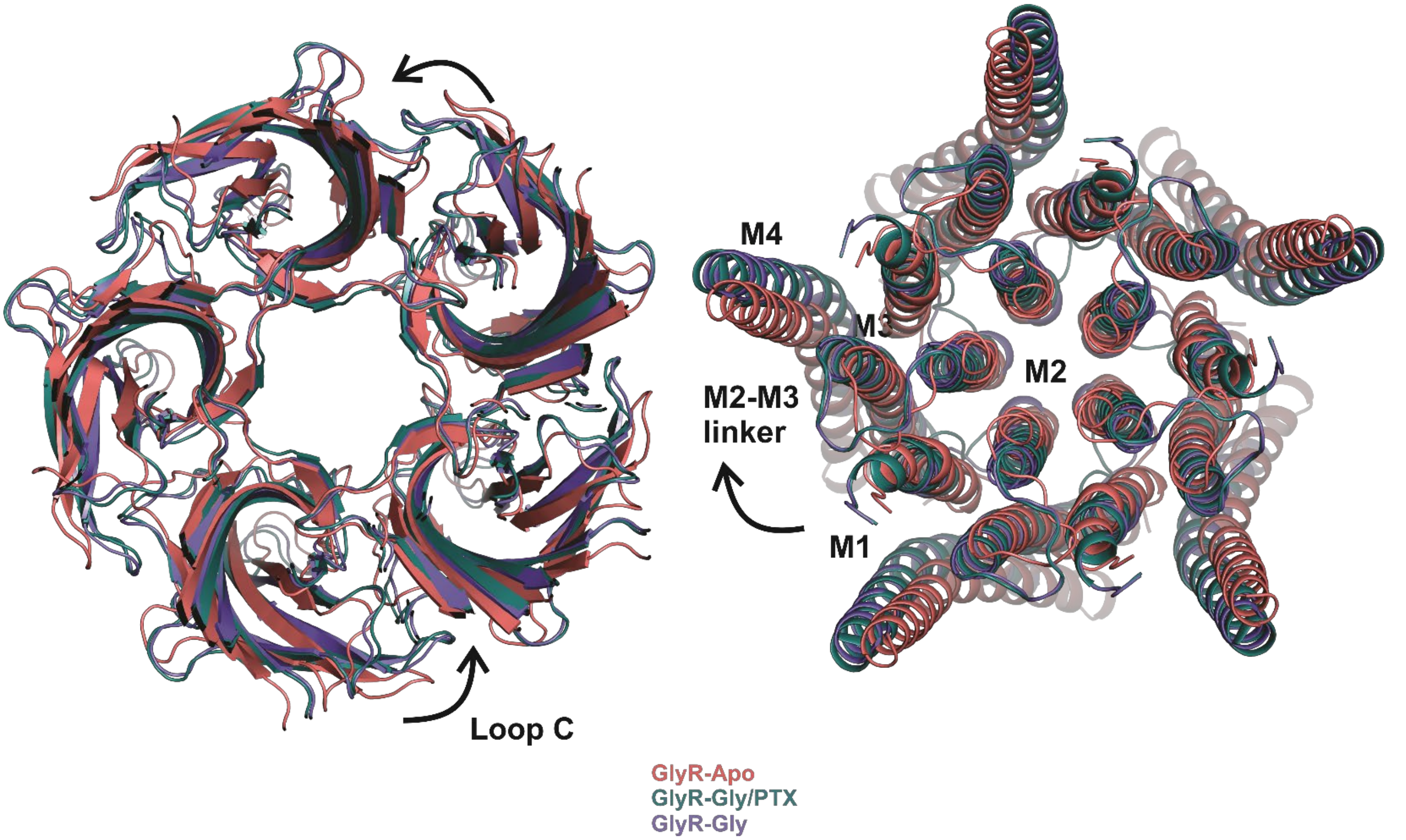


**Supplementary Figure 10.** A view of the ECD from the extracellular end upon aligning GlyR-Apo, GlyR-Gly/PTX, and GlyR-Gly (*right*). Glycine induced anti-clockwise rotation of the ECD is highlighted by black arrows in the vicinity of Loop C. A view of the TMD from the extracellular end upon aligning GlyR-Apo, GlyR-Gly/PTX, and GlyR-Gly (*left*). There is a clockwise rotation of the TMD helices with an outward expansion resembling the opening of an iris.

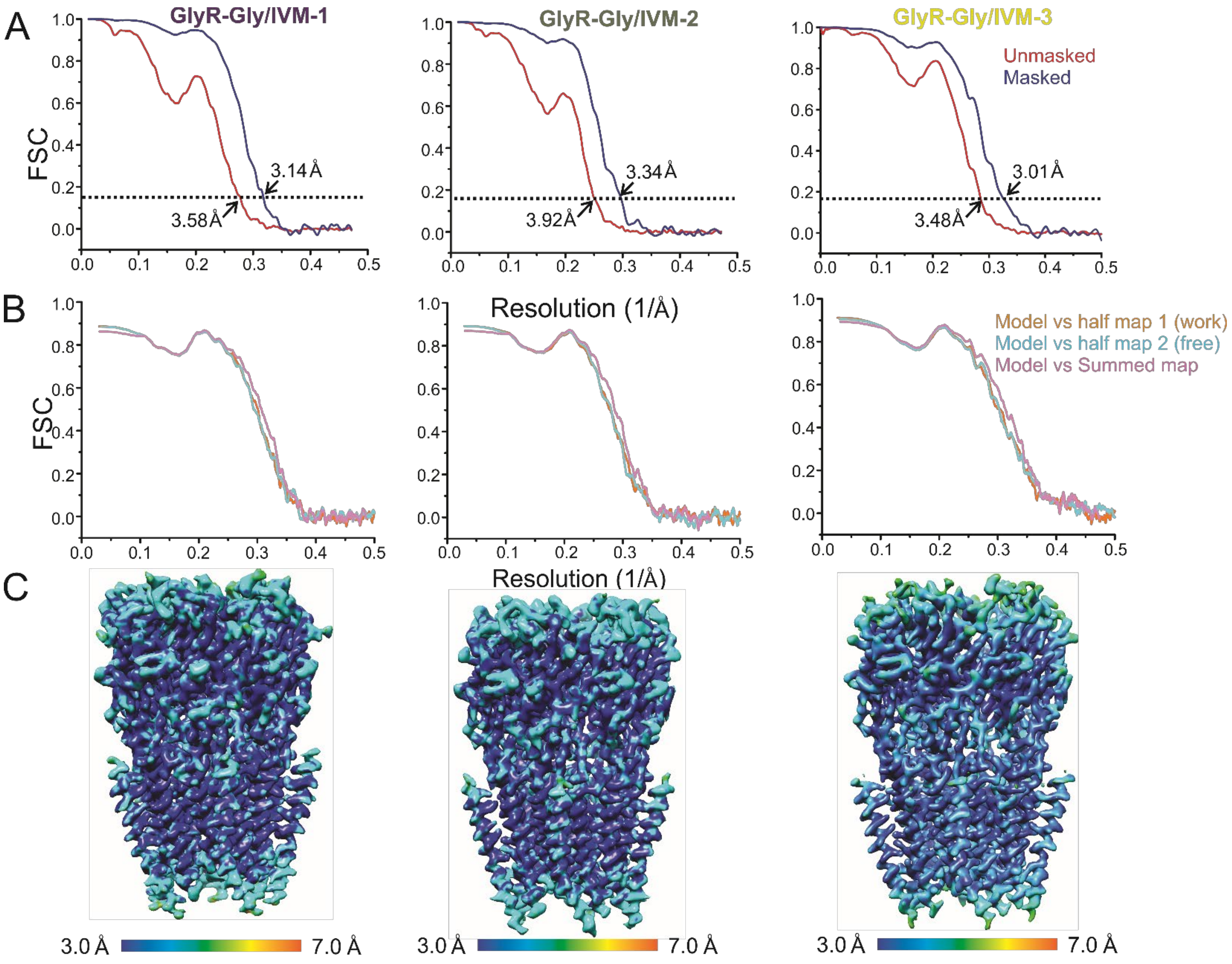


**Supplementary Figure 11. Resolution estimation and model validation for GlyR-Gly/IVM structures.** Fourier shell correlation (FSC) curves before (red) and after post-processing (blue) using gold-standard refinement in RELION 3.1 *(top)*. The dashed line represents an FSC of 0.143. For cross validation of model refinement, FSC curves of the refined model versus summed map (full dataset), refined model versus half map 1 (used during refinement), and refined model versus half map 2 (not used during refinement) *(middle)*. Side views of the 3D reconstructions colored-coded by the local resolution determined using ResMap program algorithm v1.1.5[1] *(bottom)*.

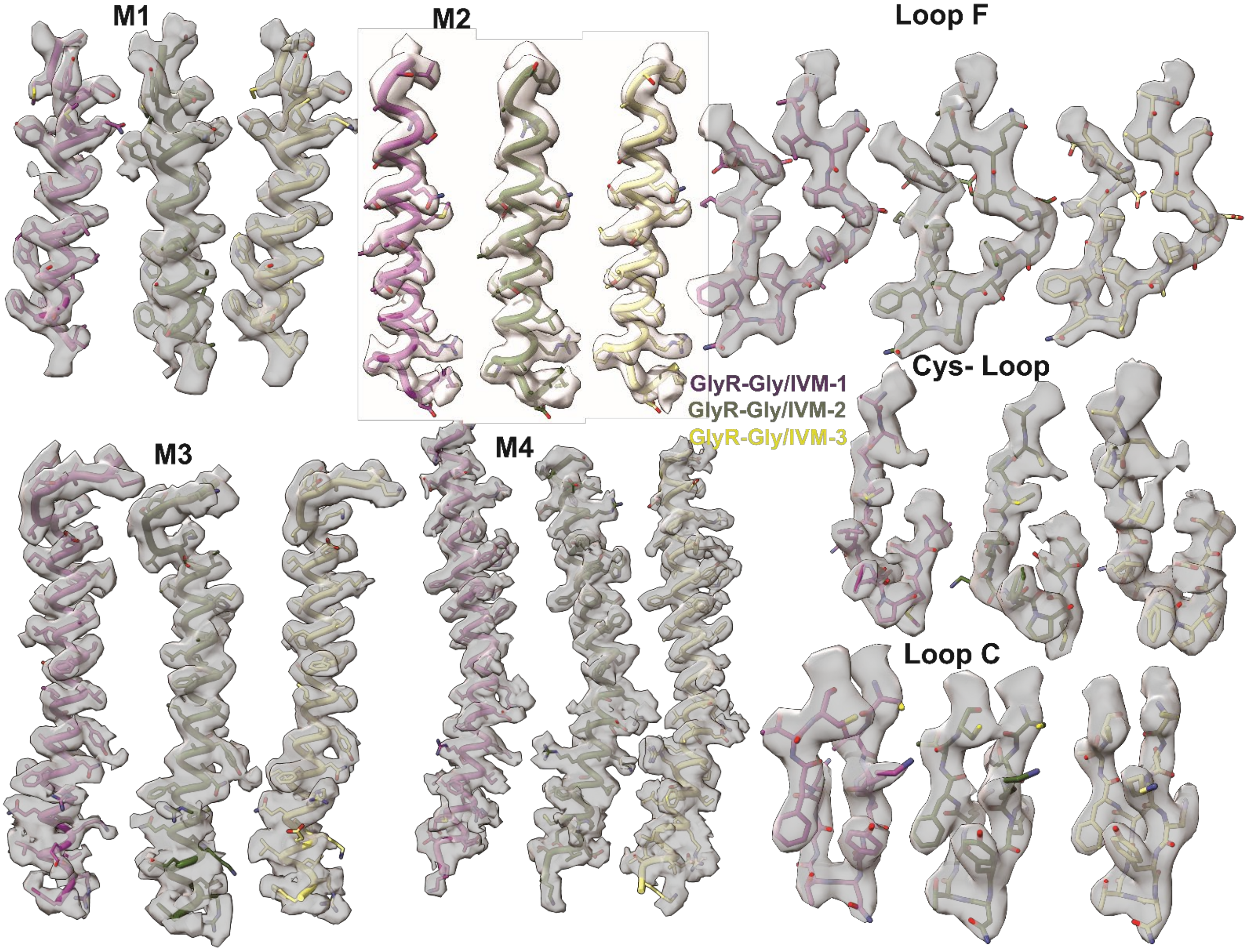


**Supplementary Figure 12. Map correlation of GlyR-Gly/IVM structures.** Validation of various regions within each of the domains of the model (shown as cartoon with stick representation for the residues) and corresponding density map are shown here. Density map for GlyR-Gly/IVM-1, GlyR-Gly/IVM-2 and GlyR-Gly/IVM-3 was contoured at a threshold of 0.0072, 0.079 and 0.0071 respectively in UCSF ChimeraX.

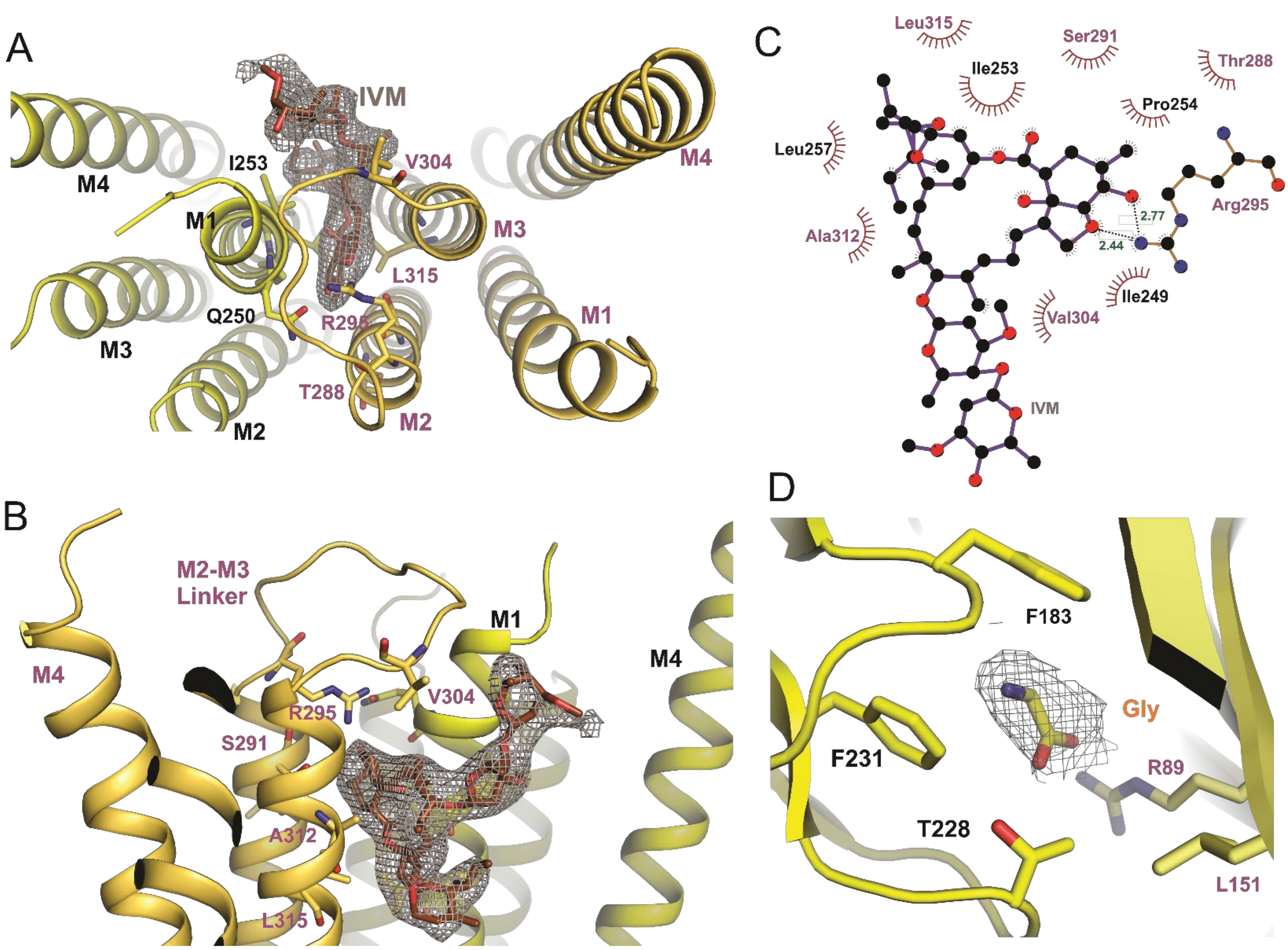


**Supplementary Figure 13. Analysis of ligand-binding sites in GlyR-Gly/IVM structures.** A) Top view of the TBD intersubunit interface with IVM binding site in the Gly-Gly/IVM-3 structure. The map of IVM density is contoured at (5σ) B) Side view of the IVM binding pocket. C) LigPlot analysis of IVM interaction with binding-site residues. D) Neurotransmitter binding pocket with glycine density contoured at (3.5σ).

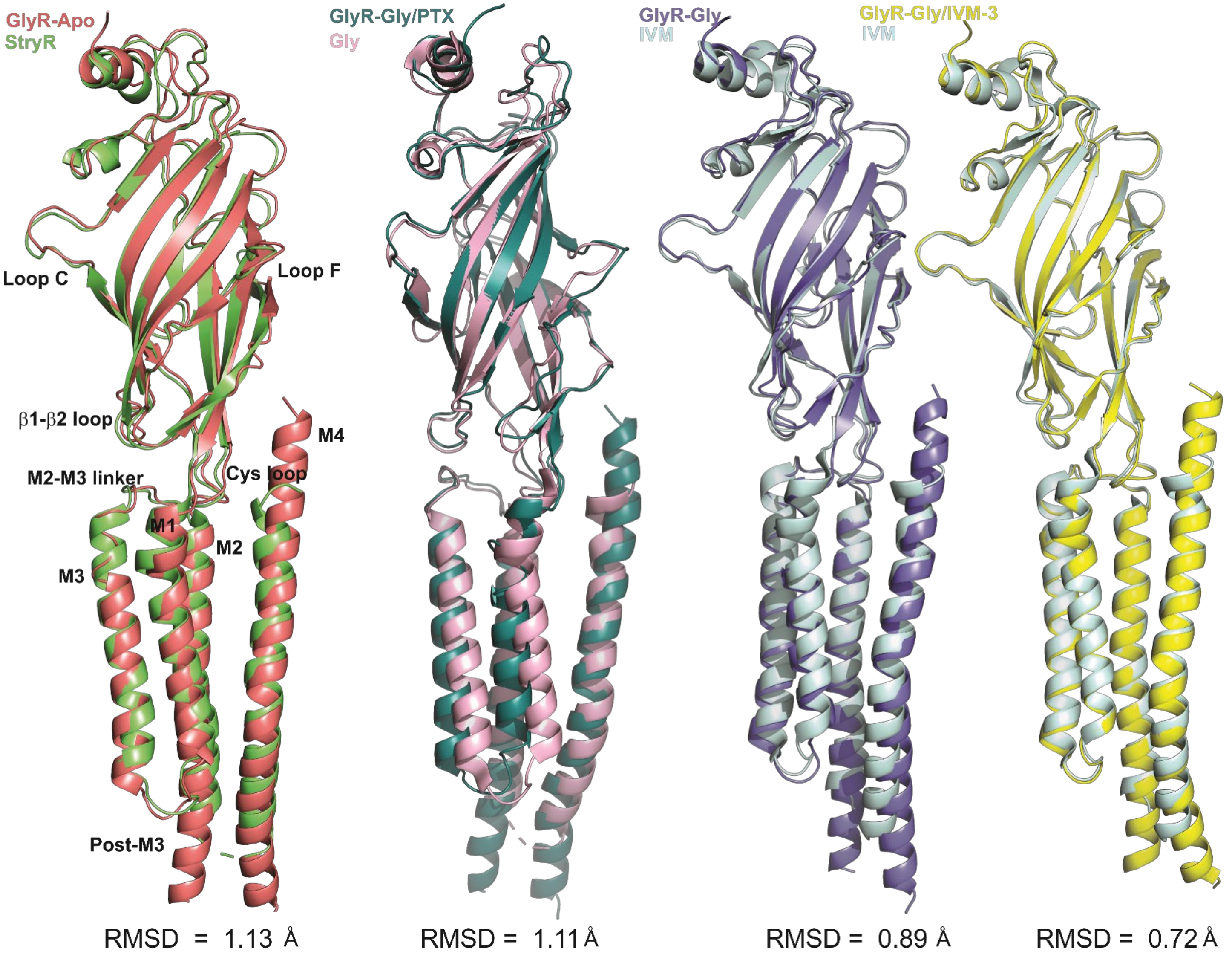


**Supplementary Figure 14. Comparison with previous GlyR cryo-EM structures.** Superposition of the single subunits of GlyR-Apo with GlyR strychnine-bound state (PDB_ID:3JAD), GlyR-Gly/PTX with GlyR Gly-bound open state (PDB_ID:3JAE) [6], and GlyR-Gly and GlyR-Gly/IVM-3 with GlyR ivermectin-bound desensitized state (PDB_ID:3JAF)[6]. The RMSD (alignment of the pentamers) were calculated to be 1.13Å, 1.11Å, 0.89Å, and 0.72 Å respectively.

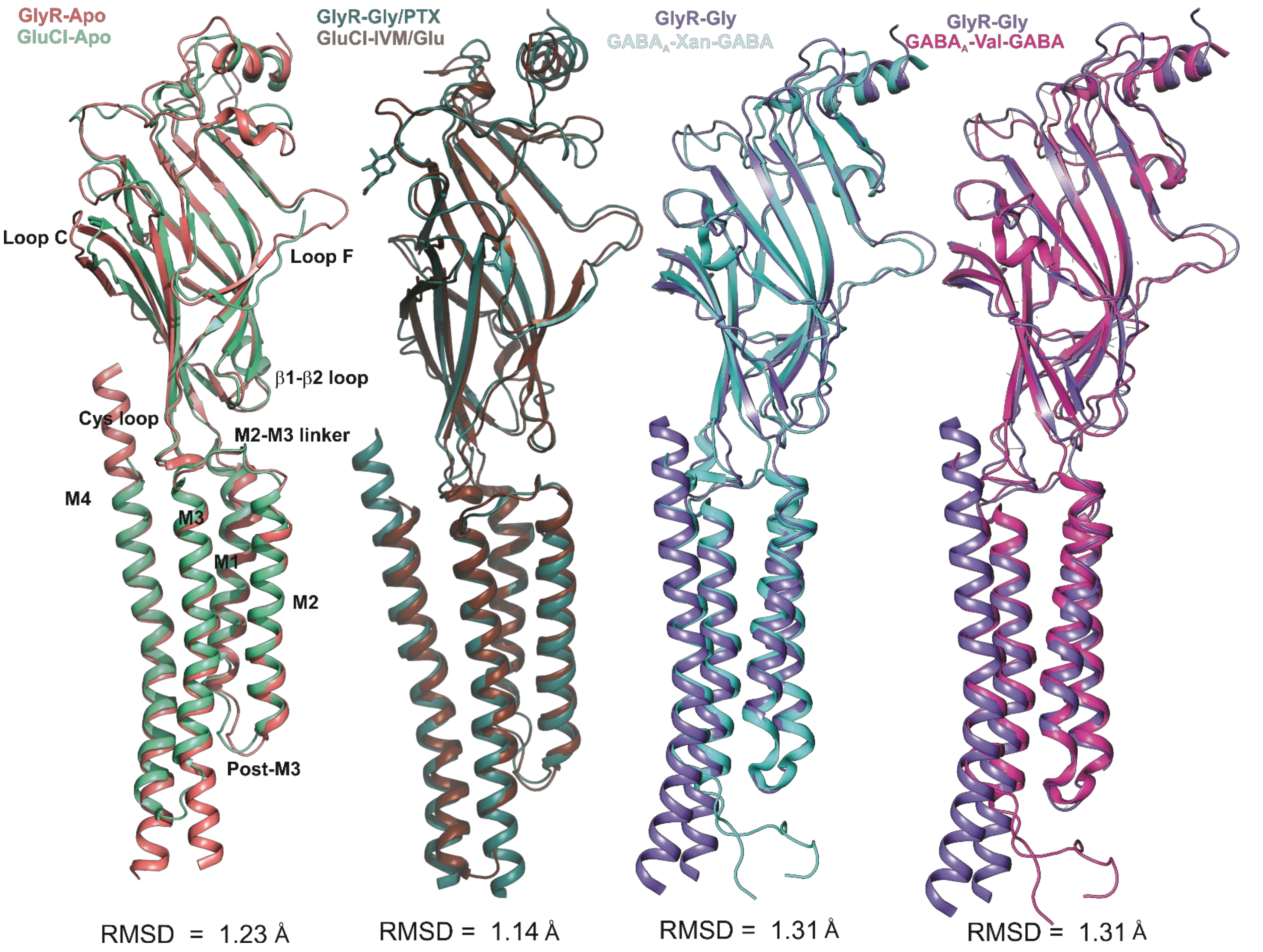


**Supplementary Figure 15. Comparison with anionic pLGIC structures.** Superposition of the single subunits of GlyR-Apo with GluCl-Apo (PDB_ID:4TNV)[7], GlyR-Gly/PTX with GluCl IVM/glutamate-bound open state (PDB_ID:3RIF)[5], GlyR-Gly with GABAA xanax/GABA-bound desensitized state (PDB_ID:6HUO)[4], GlyR-Gly with valium/GABA-bound desensitized state desensitized state (PDB_ID:6HUP)[4]. The RMSD (alignment of the pentamers) for the GluCl structures and for the α-subunit for the GABA heteromers.

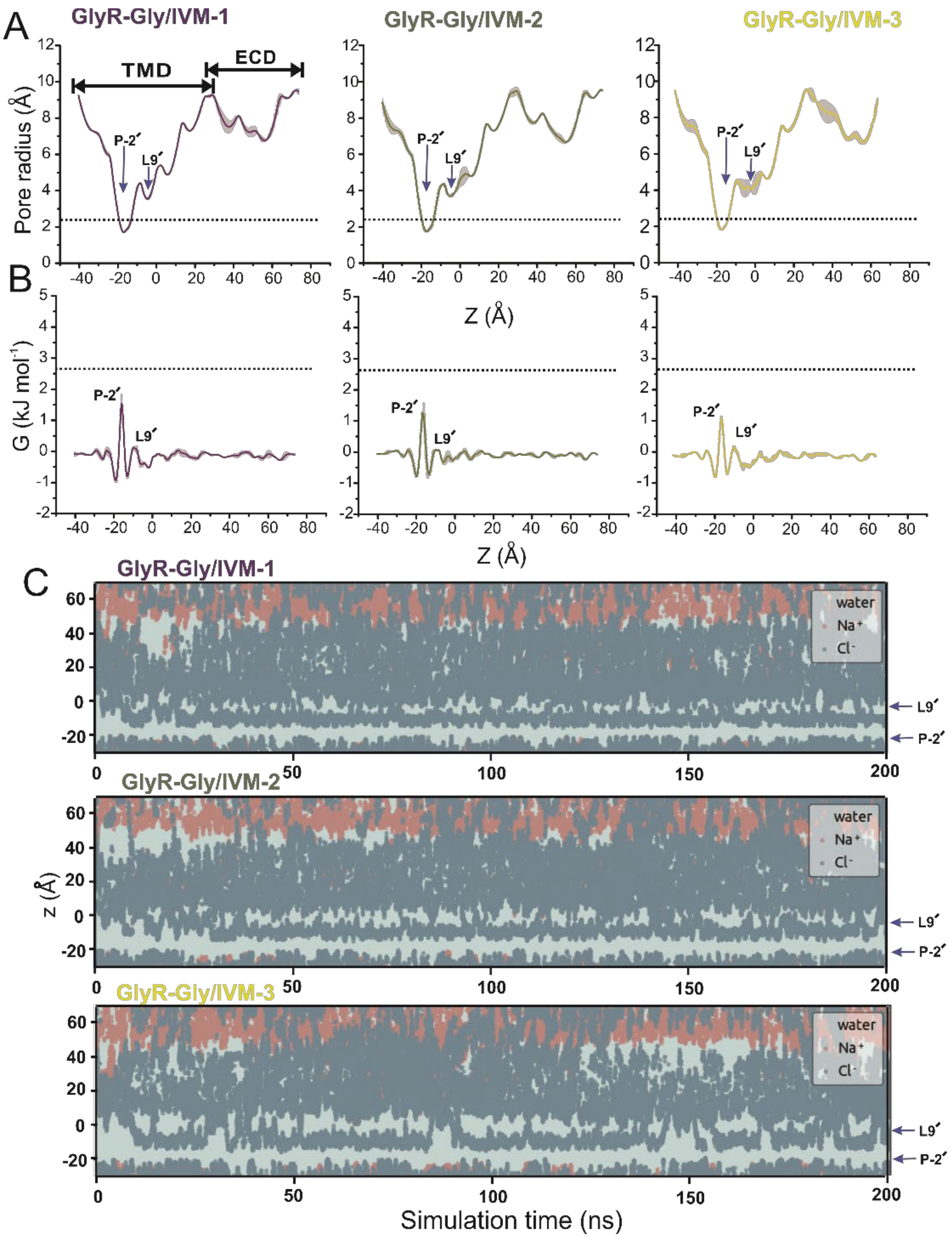


**Supplementary Figure 16**. **Molecular dynamics simulations of GlyR-Gly/IVM conformations** **A)** Mean pore radius profiles and standard deviations averaged across three independent 30 ns equilibrium simulations for GlyR-Gly/IVM states along the central pore axis. The final 20 ns of each 30 ns simulation trajectory was used to evaluate these profiles. The one-standard-deviation range between calculations (n = 3) are shown as a gray band. Major constriction sites are indicated and the dotted line denotes the radius of hydrated chloride ion. B) Corresponding mean water free energy profiles and standard deviations. Peaks in free energy profiles are highlighted. C**)** Trajectories along the pore (z) axis of water molecules and chloride ion coordinates within 5 Å of the channel axis inside the pore, in the presence of a +500 mV transmembrane potential difference (i.e. with the cytoplasmic side having a positive potential). One of five independent 200 ns replicates is shown for each structure. During these and the preceding simulations, positional restraints were placed on the protein backbone, in order to preserve the experimental conformational state whilst permitting rotameric flexibility in amino acid side chains. The energetic barriers due to the ring of Pro-2′ are at z ~ -20 Å.

Forward: 5’- GTC GAC GAG CTC GAG CTC ATG TTC GCC CTG -3’
Reverse: 5’- ATCCTCTAGTACTTCTCGACAAGCTTTTAGGTACCGTGATG -3’

**Supplementary Table 1.** Primers for cloning of GlyR into pTLN vector for oocyte expression using SacI and BamHI restriction sites.

```
>GlyRalpha1_codon_optimised
ACTAGTATGTTCGCCCTGGGTATCTACCTGTGGGAAACCATCGTGTTCTTCTCCCTGGCTGCTAGCCAGCAGGCTGC
TGCTCGCAAGGCCGCTTCCCCTATGCCTCCCAGCGAATTCCTGGACAAGCTGATGGGCAAGGTGTCCGGCTACGACG
CTCGCATCCGTCCCAACTTCAAGGGTCCACCTGTGAACGTCACTTGCAACATCTTCATCAACTCTTTCGGCTCAATC
GCCGAGACTACCATGGACTACAGGGTGAACATCTTCCTGAGACAGCAGTGGAACGACCCACGTCTGGCTTACTCTGA
ATACCCTGACGACTCACTGGACCTGGACCCCTCTATGCTGGACTCAATCTGGAAGCCAGACCTGTTCTTCGCCAACG
AGAAGGGCGCTAACTTCCACGAAGTGACCACTGACAACAAGCTGCTGAGGATCTCCAAGAACGGAAACGTGCTGTAC
AGCATCAGAATCACCCTGGTCCTGGCCTGCCCTATGGACCTGAAGAACTTCCCCATGGACGTCCAGACCTGCATCAT
GCAGCTGGAGTCCTTCGGTTACACTATGAACGACCTGATCTTCGAGTGGGACGAAAAGGGTGCTGTGCAGGTGGCTG
ACGGACTGACCCTGCCTCAGTTCATCCTGAAGGAGGAAAAGGACCTGCGCTACTGCACTAAGCACTACAACACCGGA
AAGTTCACTTGCATCGAGGCTCGCTTCCACCTGGAACGTCAGATGGGTTACTACCTGATCCAGATGTACATCCCCAG
CCTGCTGATCGTGATCCTGTCCTGGGTCAGCTTCTGGATCAACATGGACGCTGCTCCAGCTAGGGTGGGTCTGGGCA
TCACCACTGTCCTGACTATGACCACTCAGTCCAGCGGCTCTAGAGCTTCACTGCCCAAGGTGTCCTACGTCAAGGCC
ATCGACATCTGGATGGCTGTGTGCCTGCTGTTCGTCTTCAGCGCCCTGCTGGAGTACGCCGCTGTGAACTTCATCGC
TCGCCAGCACAAGGAACTGCTGCGTTTCCAGCGCCGTAGGAGACACCTGAAGGAGGACGAAGCTGGAGACGGAAGGT
TCTCTTTCGCCGCTTACGGCATGGGACCAGCCTGCCTGCAGGCTAAGGACGGAATGGCCATCAAGGGTAACAACAAC
AACGCTCCTACCTCAACTAACCCTCCTGAGAAGACCGTGGAGGAAATGCGCAAGCTGTTCATCTCTAGGGCCAAGAG
AATCGACACTGTGTCACGTGTCGCTTTCCCTCTGGTCTTCCTGATCTTCAACATCTTCTACTGGATCACCTACAAGA
TCATCCGCTCCGAAGACATCCACAAGCAGCTGGTTCCGCGTGGTAGTCATCACCATCACCATCACCATCACTAAGGT
ACC
```

**Supplementary Table 2. Codon optimized zebrafish GlyRα1 sequence**

**Supplementary Table 3**

| | GlyR-Apo | GlyR-Gly | GlyR-Gly-PTX |
|---|---|---|---|
| PDBid | **6UBS** | **6UBT** | **6UD3** |
| EMDB id | **20714** | **20715** | **20731** |
| **Data Collection and processing** | | | |
| Microscope and location | FEI Titan Krios, Frederick National Laboratory for Cancer Research | FEI Titan Krios, Frederick National Laboratory for Cancer Research | FEI Titan Krios, Stanford-SLAC Cryo-EM Center |
| Magnification | 130000 | 81000 | 81000 |
| Voltage | 300 | 300 | 300 |
| Data collection mode | super-resolution mode | super-resolution mode | Counting mode |
| Camera | K2 Summit | K3 | K2 Summit |
| Physical pixel size | 1.06 Å/pixel | 1.08 Å/pixel | 1.06 Å/pixel |
| Defocus range (μM) | -1.5 to -2.5 | -1.5 to -2.5 | -1.5 to -2.5 |
| Number of images | 2389 | 5344 | 2280 |
| Number of frames/image | 40 | 40 | 40 |
| Initial particle number | 444,921 | 466,174 | 440, 516 |
| Final particle number | 19653 | 8255 | 10, 375 |
| Symmetry | C5 | C5 | C5 |
| Resolution (unmasked, Å) | 3.89 | 4.24 | 4.2 |
| Resolution (masked, Å) | 3.33 | 3.47 | 3.51 |
| Map resolution range * | 3-7 | 3-7 | 3-7 |
| Map sharpening B-factor ($Å^2$) | -50 | -30 | -20 |
| **Refinement** | | | |
| Initial model used (PDB code) | 3JAD | 6UBS | 6UBS |
| **Composition** | | | |
| Number of atoms | | | |
| Protein residues | 1810 | 1770 | 1765 |
| Non Hydrogen atoms | 15270 | 14505 | 14346 |
| Glycan (NAG) (molecule) | 10 | 10 | 5 |
| PX4 (molecule) | 10 | 0 | 0 |
| PIO (molecule) | 5 | 0 | 0 |
| Glycine (molecule) | 0 | 5 | 5 |
| RI5 (molecule) | 0 | 0 | 1 |
| Bonds (RMSD) | | | |
| Length (Å) (# > 4σ) | 0.004 (0) | 0.007 (0) | 0.013 (27) |
| Angles (°) (# > 4σ) | 0.796 (20) | 1.506 (45) | 1.541 (56) |
| Ramachandran plot (%) | | | |
| Outliers | 0 | 0 | 0 |
| Allowed | 4.19 | 7.11 | 4.6 |
| Favored | 95.81 | 92.89 | 95.4 |
| Rotamer outliers (%) | 1.22 | 0.31 | 0 |
| Molprobity score | 1.64 | 1.77 | 1.72 |
| Molprobity clashscore | 5.31 | 5.75 | 7.27 |

* Local resolution range

## Supplementary Table 4

| | GlyR-Gly-IVM-State-1 | GlyR-Gly-IVM-State-2 | GlyR-Gly-IVM-State-3 |
|---|---|---|---|
| PDBid | **6VM0** | **6VM2** | **6VM3** |
| EMDB id | **21234** | **21236** | **21237** |
| **Data Collection and processing** | | | |
| Microscope and location | FEI Titan Krios, Frederick National Laboratory for Cancer Research | FEI Titan Krios, Frederick National Laboratory for Cancer Research | FEI Titan Krios, Case Western Reserve University |
| Magnification | 81000 | 81000 | 105000 |
| Voltage | 300 | 300 | 300 |
| Data collection mode | super-resolution mode | super-resolution mode | super-resolution mode |
| Camera | K3 | K3 | K3 |
| Physical pixel size | 1.08 Å/pixel | 1.08 Å/pixel | 0.84 Å/pixel |
| Defocus range (uM) | -1.5 to -2.5 | -1.5 to -2.5 | -1.5 to -2.25 |
| Number of images | 9700 | 9700 | 2193 |
| Number of frames/image | 40 | 40 | 40 |
| Initial particle number | 860560 | 860560 | 288811 |
| Final particle number | 19600 | 15035 | 27516 |
| Symmetry | C5 | C5 | C5 |
| Resolution (unmasked, Å) | 3.58 | 3.92 | 3.48 |
| Resolution (masked, Å) | 3.14 | 3.34 | 3.01 |
| Map resolution range * | 3-7 | 3-7 | 3-7 |
| Map sharpening B-factor (Å$^2$) | -30 | -30 | -30 |
| **Refinement** | | | |
| Intial model used (PDB code) | 6UBS | 6UBS | 6UBS |
| **Composition** | | | |
| Protein residues | 1810 | 1810 | 1810 |
| Non Hydrogen atoms | 15465 | 15355 | 15465 |
| Glycan (NAG) (molecule) | 10 | 10 | 10 |
| PIO (molecule) | 5 | 5 | 0 |
| Glycine (molecule) | 5 | 5 | 5 |
| IVM | 5 | 5 | 5 |
| Bonds (RMSD) | | | |
| Length (Å) (# > 4σ) | 0.009 (0) | 0.011 (0) | 0.011(0) |
| Angles (°) (# > 4σ) | 1.235(25) | 1.329 (20) | 1.483(30) |
| Ramachandran plot (%) | | | |
| Outliers | 0 | 0 | 0 |
| Allowed | 3.91 | 4.19 | 3.08 |
| Favored | 96.09 | 95.81 | 96.92 |
| Rotamer outliers (%) | 0 | 0 | 0.92 |
| Molprobity score | 1.64 | 1.6 | 1.51 |
| Molprobity clashscore | 6.69 | 5.64 | 5.9 |

* Local resolution range

## References


1 Kucukelbir, A., Sigworth, F. J. & Tagare, H. D. Quantifying the local resolution of cryo-EM density maps. *Nat Methods* **11**, 63-65, doi:10.1038/nmeth.2727 (2014).

2 Rao, S., Klesse, G., Stansfeld, P. J., Tucker, S. J. & Sansom, M. S. P. A heuristic derived from analysis of the ion channel structural proteome permits the rapid identification of hydrophobic gates. *Proc Natl Acad Sci U S A* **116**, 13989-13995, doi:10.1073/pnas.1902702116 (2019).

3 Baker, N. A., Sept, D., Joseph, S., Holst, M. J. & McCammon, J. A. Electrostatics of nanosystems: application to microtubules and the ribosome. *Proc. Natl Acad. Sci. USA* **98**, 10037-10041 (2001).

4 Masiulis, S. *et al.* GABAA receptor signalling mechanisms revealed by structural pharmacology. *Nature*, doi:10.1038/s41586-018-0832-5 (2019).

5 Hibbs, R. E. & Gouaux, E. Principles of activation and permeation in an anion-selective Cys-loop receptor. *Nature* **474**, 54-60, doi:10.1038/nature10139 (2011).

6 Du, J., Lu, W., Wu, S., Cheng, Y. & Gouaux, E. Glycine receptor mechanism elucidated by electron cryo-microscopy. *Nature*, doi:10.1038/nature14853 (2015).

7 Althoff, T., Hibbs, R. E., Banerjee, S. & Gouaux, E. X-ray structures of GluCl in apo states reveal a gating mechanism of Cys-loop receptors. *Nature* **512**, 333-337, doi:10.1038/nature13669 (2014).
