# Supplementary material for: Mechanisms of activation and desensitization of full-length glycine receptor in lipid nanodiscs
Source: Nat Commun. 2020 Jul 27;11:3752. doi: 10.1038/s41467-020-17364-5 (PMC7385131; doi:10.1038/s41467-020-17364-5)
Supplement: Supplementary file 3 — Description of Additional Supplementary Files [file 41467_2020_17364_MOESM3_ESM.pdf]

Description of Additional Supplementary Files

Supplementary Movie 1. Conformational changes underlying GlyR gating. A morph of the GlyR-Apo, GlyR-Gly/PTX, and GlyR-Gly structures to highlight the global conformational changes that ensues upon glycine binding in all three domains of the channel.

Supplementary Movie 2. **Trajectory of a single chloride ion (purple sphere) traveling through the GlyR-Gly/PTX structure, in the presence of a transmembrane potential difference of 500 mV.** The duration of the movie corresponds to ~20 ns of a 200 ns simulation. Water molecules within 4Å of the ion at a given instant are shown as red-and-white sticks; all other water and ions present in the system are omitted for clarity. Two subunits of the protein (semi-transparent surface) are shown in cartoon representation. Membrane lipid head-groups are colored orange.
