# Supplementary material for: Mechanisms of activation and desensitization of full-length glycine receptor in lipid nanodiscs
Source: Nat Commun. 2020 Jul 27;11:3752. doi: 10.1038/s41467-020-17364-5 (PMC7385131; doi:10.1038/s41467-020-17364-5)
Supplement: Supplementary file 6 — Reporting Summary [file 41467_2020_17364_MOESM6_ESM.pdf]

nature research

Corresponding author(s): Sudha Chakrapani

Last updated by author(s): May 4, 2020

Reporting Summary

Nature Research wishes to improve the reproducibility of the work that we publish. This form provides structure for consistency and transparency in reporting. For further information on Nature Research policies, see our Editorial Policies and the Editorial Policy Checklist.

Statistics

For all statistical analyses, confirm that the following items are present in the figure legend, table legend, main text, or Methods section.

n/a	Confirmed	
☐	☒	The exact sample size (*n*) for each experimental group/condition, given as a discrete number and unit of measurement
☐	☒	A statement on whether measurements were taken from distinct samples or whether the same sample was measured repeatedly
☒	☐	The statistical test(s) used AND whether they are one- or two-sided<br>*Only common tests should be described solely by name; describe more complex techniques in the Methods section.*
☒	☐	A description of all covariates tested
☒	☐	A description of any assumptions or corrections, such as tests of normality and adjustment for multiple comparisons
☐	☒	A full description of the statistical parameters including central tendency (e.g. means) or other basic estimates (e.g. regression coefficient) AND variation (e.g. standard deviation) or associated estimates of uncertainty (e.g. confidence intervals)
☒	☐	For null hypothesis testing, the test statistic (e.g. *F*, *t*, *r*) with confidence intervals, effect sizes, degrees of freedom and *P* value noted<br>*Give P values as exact values whenever suitable.*
☒	☐	For Bayesian analysis, information on the choice of priors and Markov chain Monte Carlo settings
☒	☐	For hierarchical and complex designs, identification of the appropriate level for tests and full reporting of outcomes
☒	☐	Estimates of effect sizes (e.g. Cohen's *d*, Pearson's *r*), indicating how they were calculated

Our web collection on statistics for biologists contains articles on many of the points above.

Software and code

Policy information about availability of computer code

Data collection: Clampfit 10.2, Latitude S (Gatan), EPU 2.6 (ThermoFisher scientific)

Data analysis:
1. Drift correction: MotionCor version 2.1.2.3
2. CTF estimation: GCTF version 1.06, ctffind 4
3. 2D and 3D Reconstruction, 3D refinement, post-processing: Relion Version 3.06, cisTEM 1.0.0 beta
4. Local resolution estimation: Resmap version 1.1.4
5. Pore profile calculation: HOLE version 3.0, Channel annotation package 0.9.1
6. Model visualization: PyMOL version 2.0.4
7. 3D volume visualization: Chimera version 1.11.2, ChimeraX 0.93
8. MRC to MTZ conversion: CCP4I version 7.0.057
9. Manual model building: Coot 0.8.9.1
10. Structure refinement: Phenix version 1.17rc5-3630
11. Electrostatic potential calculation: APBS version 1.5
12. Protein surface area and interfaces analysis: PDBePISA server v1.48
13. MD simulations: Gromacs 2018
14. Figure generation: CorelDraw version 20.1.0.708
15. Electrophysiology data analysis: Clampfit version 10.2
16. Electrophysiology traces: OriginLab Version b9.5.0.193
17. Cavity prediction: fpocket 2.0

For manuscripts utilizing custom algorithms or software that are central to the research but not yet described in published literature, software must be made available to editors and reviewers. We strongly encourage code deposition in a community repository (e.g. GitHub). See the Nature Research guidelines for submitting code & software for further information.

Data

Policy information about availability of data

All manuscripts must include a data availability statement. This statement should provide the following information, where applicable:

- Accession codes, unique identifiers, or web links for publicly available datasets
- A list of figures that have associated raw data
- A description of any restrictions on data availability

The atomic coordinates and cryo-EM maps for the GlyR-Apo, GlyR-Gly, and GlyR-Gly/PTX structures have been deposited in the Protein Data Bank and Electron Microscopy Data Bank with accession codes PDB-6UBS (EMD-20714), PDB-6UBT (EMD-20715) and PDB-6UD3 (EMD-20731), respectively. The atomic coordinates and cryo-EM maps for the GlyR-Gly-IVM-1, GlyR-Gly-IVM-2, and GlyR-Gly-IVM-3 structures have been deposited in the Protein Data Bank and Electron Microscopy Data Bank with accession codes PDB-6VM0 (EMD-21234), PDB-6VM2 (EMD-21236) and PDB-6VM3 (EMD-21237), respectively. All relevant data are available from the corresponding author upon reasonable request.

Field-specific reporting

Please select the one below that is the best fit for your research. If you are not sure, read the appropriate sections before making your selection.

[x] Life sciences [ ] Behavioural & social sciences [ ] Ecological, evolutionary & environmental sciences

For a reference copy of the document with all sections, see nature.com/documents/nr-reporting-summary-flat.pdf

Life sciences study design

All studies must disclose on these points even when the disclosure is negative.

Sample size	For electrophysiological recordings, the wild type currents were recorded from independently injected oocytes. Ligand activation, current block, and potentiation were observed in multiple oocytes (sample size indicated in the figure legend).
Data exclusions	For electrophysiological recordings, currents from unhealthy or leaky oocytes were not included in the analysis.
Replication	Electrophysiology experiments were performed on independent oocytes, from multiple different surgeries. Simulations were set up in triplicates. Number of replicates for experimental and simulation data are indicated in the legend. Structure determination was not carried out in replicates which is a standard practice in the field.
Randomization	Randomization is not applicable for structural studies. For electrophysiology experiments, oocytes were randomly used various types of ligand-application experiments.
Blinding	Blinding was not used since it is not applicable to structural studies or reported functional measurements.

Reporting for specific materials, systems and methods

We require information from authors about some types of materials, experimental systems and methods used in many studies. Here, indicate whether each material, system or method listed is relevant to your study. If you are not sure if a list item applies to your research, read the appropriate section before selecting a response.

Materials & experimental systems

n/a	Involved in the study	
[x]	[ ]	Antibodies
[x]	[ ]	Eukaryotic cell lines
[x]	[ ]	Palaeontology and archaeology
[ ]	[x]	Animals and other organisms
[x]	[ ]	Human research participants
[x]	[ ]	Clinical data
[x]	[ ]	Dual use research of concern

Methods

n/a	Involved in the study	
[x]	[ ]	ChIP-seq
[x]	[ ]	Flow cytometry
[x]	[ ]	MRI-based neuroimaging

Animals and other organisms

Policy information about studies involving animals; ARRIVE guidelines recommended for reporting animal research

Laboratory animals	1. Female Xenopus laevis were purchased from Nasco.<br>2. Sf9 cell purchased from Invitrogen.
Wild animals	N/A

Field-collected samples	The study did not involve samples collected from the field.
Ethics oversight	Dr. W. F. Boron kindly provided oocytes used in this study. We complied with all relevant ethical regulations for animal testing and research. Animal experimental procedures were approved by Institutional Animal Care and Use Committee (IACUC) of Case Western Reserve University.

Note that full information on the approval of the study protocol must also be provided in the manuscript.
